# Factors associated with malnutrition among older people in Swedish short‐term care: Poor oral health, dysphagia and mortality

**DOI:** 10.1111/idh.12832

**Published:** 2024-06-02

**Authors:** Susanne Lindqvist, Lena Olai, Patricia Hägglund

**Affiliations:** ^1^ Department of Odontology, Dental Hygienist Education Umeå University Umeå Sweden; ^2^ School of Education, Health and Social Studies Dalarna University Falun Sweden; ^3^ Department of Public Health and Caring Sciences, Family Medicine and Preventive Medicine Uppsala University Uppsala Sweden; ^4^ Department of Clinical Sciences, Speech‐Language Pathology Umeå University Umea Sweden

**Keywords:** intermediate care, mortality, oral health, risk factors, swallowing disorders, undernutrition

## Abstract

**Objectives:**

To investigate the relationship between malnutrition and potential contributing factors such as poor oral health, dysphagia and mortality among older people in short‐term care.

**Methods:**

This cross‐sectional study is a part of the multidisciplinary multicentre project SOFIA (Swallowing function, Oral health and Food Intake in old Age), which includes older people (≥65 years) in 36 short‐term care units in five regions of Sweden. Nutritional status was measured with version II of the Minimal Eating Observation and Nutrition Form (MEONF‐II), oral health with the Revised Oral Assessment Guide (ROAG), dysphagia with a water swallow test, and the mortality rate was followed for 1 year. Data were analysed using descriptive analysis and logistic regression models to calculate odds ratios for the association between malnutrition and these factors.

**Results:**

Among the 391 participants, the median age was 84 years and 53.3% were women. Mortality rate was 25.1% within 1 year in the total group, and was higher among malnourished participants than among their well‐nourished counterparts. Severe dysphagia (OR: 6.51, 95% CI: 2.40–17.68), poor oral health (OR: 5.73, 95% CI: 2.33–14.09) and female gender (OR: 2.2, 95% CI: 1.24–3.93) were independently associated with malnutrition.

**Conclusion:**

Mortality rate was higher among malnourished people than those who were well nourished. Severe dysphagia, poor oral health and female gender was predictors of malnutrition among older people in short‐term care. These health risks should be given more attention in short‐term care with early identification.

## 1 INTRODUCTION

The only essentials for human survival, besides oxygen, are energy and nutrients. An adequate nutritional status is thus a key factor for good general health among older people.^1^ Despite this, nutritional intake is often compromised in people aged ≥65 years, due to many factors; this increases the risk of malnutrition (i.e., undernutrition).^2^, ^3^ The prevalence of malnutrition risk among older people in residential care in Europe is high, with an estimated rate of up to 60% depending on diagnostic criteria.^4^, ^5^ In Sweden, the prevalence of risk for malnutrition has been estimated to 56% among older persons receiving municipal health care,^6^ and to 23% among people in short‐term care.^7^ Malnutrition in older people is considered to increase the risk of adverse clinical outcomes such as frailty, osteoporosis and mortality.^2^, ^8^ Further adverse outcomes of malnutrition are related to delayed wound healing, impaired muscle function, reduced heart and lung function and high hospitalization and readmission rates.^2^, ^3^

The causes of malnutrition in older people are complex and multifactorial. Malnutrition may originate from diseases both with inflammation (e.g., chronic obstructive pulmonary diseases, heart failure, or chronic renal failure) and without inflammation (e.g., stroke, Parkinson's disease and major neurocognitive disorder), or from age‐related weight loss due to reduced appetite and food intake caused by non‐inflammatory physiological changes such as reduced sensory function, dysphagia and skeletal muscle mass loss.^2^ The European Society of Clinical Nutrition and Metabolism (ESPEN) recommends that malnutrition is based on either (a) a low body mass index (BMI) (<18.5 kg/m^2^) or (b) on the combined finding of weight loss together with either reduced BMI (age‐specific) or a markedly reduced body mass using sex‐specific cut‐offs.^9^ Reduced BMI is considered <20 or <22 kg/m^2^ in subjects younger and older than 70 years, respectively. If a person shows signs of malnutrition, further investigation should be carried out in order to clarify the underlying causes.^10^

Several known risk factors for malnutrition have increased in recent decades.^2^, ^3^, ^8^ Risk factors for malnutrition in older people with the most prominent evidence include previous hospital stay, low appetite, eating dependency, e.g., needs help with the transportation of food/liquid from the plate to the mouth or the with preparation of it, low physical function and poor self‐perceived health.^3^, ^11^ Other factors that may contribute to malnutrition in older people are polypharmacy, cognitive impairment, frailty, poor oral health and dentition, dysphagia, social isolation and poverty.^2^, ^3^, ^11^

Regarding oral health, there is considered to be a dual association between nutrition and oral health status.^12^ Poor nutritional status with inadequate intake of micronutrients and macronutrients can increase the risk of oral health problems such as caries, gum disease, xerostomia (subjective sensation of dry mouth) and hyposalivation (objectively measured).^13^ Poor oral conditions may also affect chewing ability and lead to poor masticatory function, which in turn may influence dietary intake and increase the risk of malnutrition.^12^, ^14^ Good oral health is considered to include the retention of at least 20 natural teeth, which is a marker for functional natural dentition. The positions of the remaining teeth and occluding pairs are also important for good oral function.^14^ Loss of occluding tooth areas reduces the masticatory function, indicating that the number of occluding tooth areas is a crucial factor in masticatory function.^15^ At least four occluding areas have been identified as necessary to provide a stable and functional dentition with good chewing ability.^15^ However, there is only limited research on the impact of occluded areas on nutritional status.

Another factor that might contribute to malnutrition is dysphagia (i.e., difficulty in swallowing).^16^ Despite some conflicting evidence on the effect of dysphagia on malnutrition,^2^ studies have identified an ineffective swallowing with residual in the oral cavity and pharynx as a prognostic factor for malnutrition.^3^, ^16^ The impact of severe dysphagia and aspiration of saliva, food, fluid, or medicine to the lungs has been less investigated. Hypothetically, even a person who aspirate might have a nutritional deficit if accurate nutritional treatment with enteral or parenteral nutrition has not been established.

The number of older people above 80 years of age in Sweden is increasing,^17^ whereas the ability to perform activities in daily living decreases in older age resulting in higher need for care assistants. One form of nursing care is short‐term care. Short‐term care is a relatively unexplored care context where many older people stay either after being discharged from the hospital to recover or while waiting for a permanent nursing home placement. Most studies on short‐term care are retrospective studies investigating different health aspects from register data^6^ or its effectiveness in reducing hospital re‐admission.^18^ To be able to improve the health of older people in short‐term care, there is a need for knowledge of prognostic factors that may have an impact. Awareness of predictive factors for malnutrition is thus a first step towards adequate management and intervention. The specific aim of the present study was to investigate the relationship between malnutrition, poor oral health including number of teeth and occluded areas, severe dysphagia and mortality among older people in short‐term care.

## 2 STUDY POPULATION AND METHODOLOGY

### 2.1 Design and setting

This descriptive cross‐sectional study is a part of the multidisciplinary multicentre project SOFIA (Swallowing function, Oral health and Food Intake in old Age).^19^ The study was approved by the Regional Ethics Review Board, Uppsala University, Sweden (ref: 2013/100/3), conducted following the STROBE guidelines^20^ and was performed in accordance with the Helsinki Declaration.^21^ The study was retrospectively registered with Clinical Trials.gov on July 4, 2016 (identifier: NCT02825927). Ethical principles were followed, including informed consent, confidentiality and the right to withdraw from participation at any time without giving a reason. The study was performed in 36 short‐term care units in five regions of Sweden, both rural and urban. Short‐term care provides nursing care for periods of days to months for people who are, for example, undergoing rehabilitation, recovering after a hospital discharge, waiting for nursing home placement, respite care, or end‐of‐life care.^17^

### 2.2 Participants

Eligible participants were those who fulfilled the following inclusion criteria: ≥65 years old, staying for at least 3 days, able to understand Swedish and able to participate in clinical assessments. Older people in end‐of‐life care or with moderate or severe cognitive impairment based on medical records and subjectively judge by the responsible nurse at each short‐term care facility were excluded. Of the 931 available people in the short‐term care units, 477 did not fulfil the inclusion criteria and 63 (13.2%) declined to participate. Reasons for exclusion were palliative care (*n* = 61), insufficient cognitive capacity (*n* = 309) and having been admitted for less than 3 days, being younger than 65 years, or being unable to communicate in Swedish (*n* = 107). A total of 391 older people were finally included in the study.

### 2.3 Procedure

All participants provided their written informed consent. For each participant, socio‐demographic and medical data were collected from care documentation and self‐reports, including age, sex, height, weight, mild cognitive impairment, number of chronic diseases, multimorbidity and education level. Individual height and weight were used to calculate the body mass index (BMI); the BMI was defined as low <20 (age ≤69 years) or <22 (age ≥70 years) or as normal/high if above 20/22 (age ≤69 years/≥70 years).^9^ Mild cognitive impairment was based on medical records and was judged subjectively by the responsible nurse at each short‐term care facility. Multimorbidity was defined as three or more diagnoses that involved a minimum of three different organs/organ systems.^22^ The responsible nurse at each short‐term care unit also assessed the participants' functional status (i.e., care dependency) using the modified Katz Index of Activities of Daily Living (Katz‐ADL).^23^, ^24^ In this assessment, participants receive a ‘yes’ or ‘no’ score based on whether they exhibit control in bathing, dressing, toilet‐related tasks, transferring, continence and feeding. The Katz‐ADL ranges from A to G, A indicates independence (score yes in all six functions), B to D indicates moderate dependency and E to G indicates total dependency. All participants were also followed prospectively for 1 year from inclusion to assess mortality rate using the Swedish Death Register. The procedure for evaluating nutritional status, oral health and swallowing ability is described below under Section 2.4.

### 2.4 Instruments

#### 2.4.1 Assessment of the nutritional status, oral health and swallowing ability

Before the study began, eight registered dental hygienists (RDHs) and one speech‐language pathologist (SLP) were trained and calibrated in examining and performing assessments of oral health and swallowing function. All assessments were conducted in the short‐term care facilities. The RDHs carried out oral assessments with a mouth mirror and flashlight,^19^ and both the RDHs and the SLP assessed swallowing function. At each short‐term care facility, a trained nurse responsible for the participant assessed the risk of malnutrition.

#### 2.4.2 Nutritional status

This study focused on undernutrition in older people and the present article refers to undernutrition as malnutrition since these two terms are often used interchangeably. Risk of malnutrition was assessed with version II of the Minimal Eating Observation and Nutrition Form (MEONF‐II),^25^ which estimates risk on the basis of unintentional weight loss, low BMI/short calf circumference, eating difficulties (food intake, chewing/swallowing, decreased energy/appetite) and clinical signs of malnutrition. The MEONF‐II score ranges from 0 to 8; a score of 0–2 indicates no or low risk of malnutrition, 3–4 a moderate risk of malnutrition and ≥5 a high risk of malnutrition. The cut‐off ≥3 has been observed to be an optimal cut‐off for any risk of malnutrition^26^ and was therefore chosen to define the risk for malnutrition in the current study.

#### 2.4.3 Oral health

Oral health was assessed with the Revised Oral Assessment Guide (ROAG).^27^ ROAG is a valid instrument with good reliability.^27^, ^28^ The ROAG include eight categories: voice, lips, mucous membranes, tongue, gums, teeth/dentures, saliva and swallowing sensation (e.g., pain or dryness when swallowing saliva). Each category was described and rated from healthy (score 1) to severe (score 3). The total score ranged from 8 (healthy) to 24 (severe oral health problems). Participants were categorized as having good oral health (score of 8) or poor oral health (score of >8). Number of teeth was categorized as ≥10 versus <10 and number of occluding areas was categorized as ≥4 versus ≤3. The number of occluding areas was defined as opposing pair of maxillary and mandibular teeth, so the maximum of occluding areas in 28‐tooth definition was 14.

#### 2.4.4 Swallowing ability

First, a teaspoon test was performed to identify participants with major risk of aspiration (i.e., severe dysphagia).^29^ The participant was given three teaspoons of water to swallow and if aspiration signs (i.e., cough or wet/gurgling voice) were observed then the participant was identified as having a high risk of severe dysphagia. Cough and voice change after swallowing indicate water penetrating the larynx or even being aspirated into the trachea.

### 2.5 Statistical analyses

Descriptive data are presented as percentages. Comparisons were made using the chi‐squared test for categorical variables. Logistic regression was used to calculate odds ratios (ORs) with 95% confidence intervals (CIs) for the association between potential predictors and malnutrition. The models were constructed using associated factors likely to be judged as potential predictors based on the current literature. *p* < 0.05 was considered statistically significant. All statistical analyses were performed using version 29 of the IBM SPSS software package.

## 3 RESULTS

### 3.1 Participants

Data collection was performed between October 2013 and February 2016. In total 391 older people were included from 36 short‐term care units and screened for nutritional status, oral health and swallowing ability. Of these, 209 were women (median age 85 years, range 65–100 years) and 182 were men (median age 81 years, range 65–98 years). Nutritional status was assessed in 390 participants, oral health in 391 participants and swallowing in 385 participants. The reasons for admission to short‐term care and the characteristics of the participants are shown in Table 1. Of the 391 participants, 98 (25.1%) died within 1 year.

**TABLE 1:** Characteristics of the participants.

Variables	Baseline (*n* = 391)
Median age [IQR]	84.0 [78.0–89.0]
65–74 years	63 (16.1)
75–84 years	149 (38.1)
≥85 years	179 (45.8)
Sex	
Men	182 (46.5)
Women	209 (53.5)
Reason for short‐term care	
Respite care	76 (19)
Acute short‐term care	70 (18)
Recovery after hospitalization	58 (15)
Rehabilitation	50 (13)
Waiting for care placement	33 (8)
Further assessment	26 (7)
Unknown reason	78 (20)
BMI^a^	23.9 [20.8–27.2]
Normal or high	268 (68.5)
Low	123 (31.5)
Multimorbidity^b^	
No	185 (47.3)
Yes	206 (52.7)
Katz‐ADL (*n* = 384)	
A: Independent	28 (7)
B–D: Partly dependent	164 (43)
E–G: Completely dependent	192 (50)
Cognition	
Normal cognition	344 (88.0)
Mild cognitive impairment	47 (12.0)
Education (*n* = 386)	
Compulsory school	251 (64.2)
Upper secondary school	99 (25.3)
Higher education	36 (9.2)
ROAG score (*n* = 391)	10.0 [8.0–12.0]
Good oral health (score 8)	100 (25.6)
Poor oral health (score >8)	291 (74.4)
Number of teeth (*n* = 388)	
Edentulous	73 (19)
1–19	148 (38)
20–32	167 (43)
Occluded teeth areas^c^ (*n* = 368)	
≥4	238 (65)
≤3	130 (35)
Severe dysphagia (*n* = 378)	24 (7)

*Note*: Data are given as number of participants, *n* (%) or median [IQR]. Regarding missing data, the prevalence is based on complete cases for the specific variable in each analysis.

Abbreviations: BMI, body mass index; Katz‐ADL, Katz Index of Activities of Daily Living; ROAG, Revised Oral Assessment Guide.

^a^ Low BMI was defined as <20 (age <70) or <22 (age ≥70) and normal or high BMI was defined as ≥20 (age <70) or ≥22 (age ≥70).

^b^ Defined as three or more diagnoses in three different organs/organ systems.

^c^ Defined as total number of occluded teeth areas per participant.

### 3.2 Associations with malnutrition

The associations between malnutrition and other factors are presented in Table 2. A significantly higher proportion of older people who were identified as malnourished died within 1 year (40%) compared to those who were well‐nourished (21%; *p* < 0.001). Further associations were seen between risk of malnutrition and poor oral health (*p* < 0.001), severe dysphagia (*p* < 0.001) and sex (*p* < 0.007). No associations were found between risk of malnutrition and age, mild cognitive impairment, functional status, multimorbidity, educational level, the number of teeth or the number of occluded areas.

**TABLE 2:** Associations between risk of malnutrition and health‐related factors among older people (≥65 years) in short‐term care.

Characteristic	No or low risk of malnutrition *n* = 299 (77)	Risk of malnutrition *n* = 91 (23)	*p*‐Value^a^
Sex			
Men	150 (50)	31 (34)	0.007*
Women	149 (50)	60 (66)	
Age			
65–74	48 (16)	15 (16)	0.991
75–84	114 (38)	35 (39)	
≥85	137 (46)	41 (45)	
Mild cognitive impairment			
No	260 (87)	83 (91)	0.275
Yes	39 (13)	8 (9)	
Katz‐ADL (*n* = 384)			
A: Independent	23 (8)	5 (5)	0.607
B–D: Partly dependent	127 (43)	37 (41)	
E–G: Completely dependent	143 (49)	49 (54)	
Multimorbidity			
No	147 (49)	38 (42)	0.215
Yes	152 (51)	53 (58)	
Education level (*n* = 385)			
Comprehensive school	191 (64)	59 (68)	0.796
Gymnasium/high school	78 (26)	21 (24)	
Higher education	29 (10)	7 (8)	
Deceased within 1 year (*n* = 390)			
No	237 (79)	55 (60)	<0.001**
Yes	62 (21)	36 (40)	
Oral health			
Good	92 (31)	8 (9)	<0.001**
Poor	207 (69)	83 (91)	
Number of teeth^b^ (*n* = 388)			
≥10	109 (37)	28 (31)	0.342
<10	189 (63)	62 (69)	
Number of occluded areas^c^ (*n* = 367)			
≥4	186 (66)	51 (61)	0.399
≤3	97 (34)	33 (39)	
Severe dysphagia^d^ (*n* = 378)			
No	282 (97)	72 (84)	<0.001**
Yes	10 (3)	14 (16)	

*Note*: Statistical significance *p < 0.05, **p < 0.001. Data are given as number of participants, *n* (%). Regarding missing data, the prevalence is based on complete cases for the specific variable in each analysis.

^a^ Chi‐squared test.

^b^ Defined as total number of teeth in each jaw (upper and lower).

^c^ Defined as total number of occluded teeth areas per participant.

^d^ Defined as failing a teaspoon test.

### 3.3 Predictors of malnutrition

The results from the unadjusted and adjusted models regarding predictors of malnutrition are shown in Table 3. In the adjusted model, the highest odds of being malnourished were seen for severe dysphagia; participants with severe dysphagia showed 6.5 times higher odds of being malnourished than those with normal swallowing (OR: 6.51, 95% CI: 2.40–17.68; *p* < 0.001; Table 3). Participants with poor oral health had 5.7 times higher odds of being malnourished than those with good oral health (OR: 5.73, 95% CI: 2.33–14.09; *p* < 0.001; Table 3). The adjusted model also revealed that women had 2.2 times higher odds of being malnourished than men (OR: 2.2, 95% CI: 1.24–3.93; *p* = 0.007; Table 3).

**TABLE 3:** Associated factors (potential predictors) of malnutrition among older people (≥65 years) in short‐term care.

Variables	OR (95% confidence interval)			
	Unadjusted^a^	*p* Value	Adjusted^b^	*p* Value
Age (ref. 65–74)				
75–84	0.98 (0.49–1.96)	0.960	0.97 (0.44–2.14)	0.930
≥85	0.96 (0.49–1.88)	0.900	0.79 (0.35–1.76)	0.556
Sex (ref. male)	1.95 (1.20–3.18)	0.008	2.21 (1.24–3.93)	0.007
Functional status^c^ (ref. independent)				
Partly dependent	1.34 (0.48–3.77)	0.579	1.02 (0.31–3.26)	0.980
Totally dependent	1.58 (0.57–4.38)	0.382	0.86 (0.27–2.74)	0.796
Cognitive impairment (ref. normal cognition)	0.64 (0.29–1.43)	0.278	0.44 (0.15–1.27)	0.128
Multimorbidity (ref. <3 diagnoses)	1.35 (0.84–2.17)	0.216	1.46 (0.85–2.53)	0.173
Oral health (ref. good)	4.61 (2.14–9.92)	<0.001	5.73 (2.33–14.01)	<0.001*
Number of teeth (ref. ≥10)	1.28 (0.77–2.12)	0.342	1.31 (0.72–2.40)	0.380
Number of occluded areas (ref. ≥4)	1.24 (0.75–2.05)	0.400	1.20 (0.66–2.18)	0.550
Severe dysphagia (ref. normal swallowing)	5.48 (2.34–12.85)	<0.001	6.51 (2.40–17.68)	<0.001*

*Note*: Statistical significance *p < 0.001. For the logistic regression models (univariable and multivariable), the reference categories were: male sex, age 65–74, independent functional status, normal cognition, <3 diagnoses from 3 different organs/organ systems, good oral health, ≥10 teeth, ≥4 occluded teeth areas and normal swallowing.

Abbreviation: OR, odds ratio.

^a^ Univariable model.

^b^ Multivariable model.

^c^ Functional status was based on the Katz Index of Activities of Daily Living (Katz‐ADL). Independent refers to Katz‐ADL score A, partly dependent to Katz‐ADL score B–D and totally dependent to Katz‐ADL score E–G.

## 4 DISCUSSION

This study investigated the relationship between malnutrition, poor oral health, severe dysphagia and mortality among people aged 65 years and older in 36 Swedish short‐term care units. We found that poor oral health, severe dysphagia, female gender and death within 1 year were all associated with malnutrition among these older people. These results will be further discussed below in relation to previous literature, clinical application and future research recommendations.

In the present study cohort, a significantly higher proportion of older people who were identified as malnourished died within 1 year (40%) compared to those who were well‐nourished (21%). The pronounced relationship between malnutrition and mortality has been observed in several studies.^8^, ^30^, ^31^ Borgström Bolmsjö et al.^31^ found that malnourished older people in Swedish nursing care had significantly lower survival rate compared to well‐nourished residents. Similarly, in a study by Söderström et al.^8^ among older people in Swedish hospital care, malnutrition was associated with increased mortality regardless of the cause of death. This association between malnutrition and mortality highlights the need for proper nutritional screening among older people in nursing care and more specific in short‐term care in order to ensure accurate management and minimize complications.

The multivariable model showed that female gender, poor oral health and severe dysphagia were independent predictors of malnutrition. The most prominent predictor of malnutrition was severe dysphagia; participants with this condition showed 6.5 times higher odds of being malnourished than those with normal swallowing, indicating a moderate effect size.^32^ Previous studies have shown similar results regardless of the clinical setting and type or characteristics of the dysphagia. Serra‐Prat et al.^33^ reported that dysphagia in independently living older people was a risk factor for malnutrition and lower respiratory tract infection. Carrion et al.^30^ found that dysphagia was an independent risk factor for malnutrition, with an OR of 12.6 (CI: 7.49–21.2) among 1662 older people (≥70 years) admitted with an acute disease to a general hospital.

In the literature, dysphagia has been described as potentially leading to malnutrition through several pathological mechanisms, with reduced dietary intake due to impaired chewing and swallowing ability^34^ that might be disease‐related (e.g., dysphagia following a stroke). On the other hand, malnutrition might cause or exacerbate dysphagia. For example, inadequate nutrient intake can negatively impact immune response in older people, which could increase the likelihood of contracting diseases and further worsen the nutritional status.^35^ Suboptimal nutritional status might also increase the risk of developing sarcopenia (i.e., loss of muscle mass and strength), which can affect the swallowing muscles and cause sarcopenic dysphagia.^36^ Residents in short‐term care that are frail and malnourished might thus also have an increased risk of developing sarcopenic dysphagia.

Poor oral health was also found to be associated with and a predictor of malnutrition in the present study; the participants with poor oral health had 5.7 times higher odds of being malnourished than those with good oral health. This result indicates a moderate effect size.^32^ This result is in line with a previous study showing that older people with poor oral health have poorer nutritional status than those with good oral health.^33^ Similarly, in a Swedish study by Lindmark et al.^37^ among nursing home residents, a correlation was observed between poor oral health and malnutrition with approximately one‐third of the malnourished residents having oral problems. These findings might be explained by the close interaction between malnutrition and poor oral health.^2^, ^3^ While immune system impairment due to malnutrition is closely linked to an increased risk of developing oral conditions such as gum disease, periodontal disease and oral candidiasis, poor oral health might also affect nutritional intake and hence lead to malnutrition.^12^ In Swedish short‐term care, poor oral health has been reported in 34%–70% in older people based on ROAG assessment, with an higher estimate when oral health was assessed prospectively by dental hygienist.^6^, ^38^ Poor oral hygiene is thus both prominent and a known risk factor for malnutrition among older people in short‐term care,^38^ and so it is important to maintain good oral health in order to improve nutritional intake.^39^ Poor oral health with food debris and plaque can also be an indication of dysphagia,^40^ emphasizing that there is a close relationship between poor oral health, malnutrition and dysphagia. However, factors not associated with malnutrition in our study, were number of teeth and occluding areas. One possible explanation for these findings is that the participants in the current study, to a high degree, had 20 teeth or more and four or more occluded areas and thus are considered to have a good chewing ability.^15^ These findings also align with results from Nishio et al.^41^

Another predictor for malnutrition in our cohort was female gender, with women having 2.2 times higher odds of being malnourished than men. Although women were seen to have higher risk of malnutrition compared to men, this result is considered a small effect size.^32^ In a meta‐analysis of longitudinal studies by Fávaro‐Moreira,^3^ women were not seen as being at greater risk of malnutrition. Nevertheless, like the current study several other studies have shown a higher prevalence of malnutrition among women than among men.^4^, ^42^, ^43^ Reasons for gender differences have been related to social and economic circumstances, older age, a greater likelihood of being widowed, gender inequality and/or underlying differences in physiology.^42^ These potential factors that could explain the small effect of gender difference was however not further investigated in the current study. Further longitudinal studies with high‐quality design are needed to investigate the potential sex effect on malnutrition.

In our study, neither age nor advanced age (≥85 years) was a predictor of malnutrition. It has been suggested that malnutrition might not be caused by age itself, but rather by the gradual deterioration of health status and body function (i.e., frailty) caused by aging.^9^, ^44^ However, frailty is a challenging parameter to assess, since it is considered to be multidimensional. Functional decline can be seen as a measurement of frailty. Conflicting observations are seen regarding the effect of physical performance and activities of daily living on malnutrition. Some studies have identified reduced functional status as a risk factor for malnutrition,^45^ whereas others found no association.^34^, ^46^ The diversity in observation has been suggested to be related to differences between the studies regarding mean age and percentage of women. A higher mean age (85.8 years) and percentage of women (71%) was observed in a Swedish study by Mamhidir et al.,^45^ where functional decline was seen as a risk factor for malnutrition. The present study found an association between female gender and malnutrition but not between functional decline and malnutrition. The median age in our cohort was high (84 years) and thus more similar to the cohort of Mamhidir et al.^45^ This speaks against age as an influencing factor on functional decline and thus on malnutrition, but highlights the impact of gender. However, a comparison between studies is difficult due to the use of different assessment tools to measure both malnutrition and functional ability.

Cognitive impairment is another common risk factor for malnutrition highlighted in the literature^3^ that was not prominent in our study. The lack of significance for cognitive impairment might reflect the fact that this study only included participants with no or mild cognitive impairment. This is a relevant aspect, since cognitive, functional and social disability is more pronounced in the late stage of major neurocognitive disorder.^47^

The residents in short‐term care are often considered frail, which is supported by a recent study among older people receiving municipal health care in southern Sweden.^6^ The presence of multiple health risks, such as both malnutrition and poor oral health, is more common in residents in short‐term care than in nursing homes. These findings together with the findings of our study; higher mortality rate among malnourished people in short‐term care and severe dysphagia and poor oral health being predictors of malnutrition, emphasize the need for better care, management and identification of these health risks in this specific care setting. Screening could be a first step to minimize further complications such as frailty, comorbidity and premature death.

### 4.1 Methodological considerations

As with all research this study has its strengths and limitations. The high participation rate (86%) and the prospective design regarding mortality and wide demographical distribution are strengths. All assessments were also performed by trained assessors of an interprofessional nature: dental hygienists, a SLP and nurses. We did not investigate the cause of malnutrition; for example, whether the risk of malnutrition was disease‐related (e.g., inflammatory or non‐inflammatory cause) or age‐related (e.g., loss of muscle mass), which might impact the interpretation of the results. Different causes might have different isolated predictors. The present study only investigated the effect of multimorbidity on malnutrition and not the specific diseases. The small number of participants in some subgroups (e.g., mild cognitive impairment and severe dysphagia) also needs consideration. The results would also be more generalizable to all residents in short‐term care if (a) a complete Mini Mental State Examination (MMSE)^48^ had been conducted instead of assessing mild cognitive impairment based on medical records and subjective judgements. However, adding MMSE to the full study protocol could have made the examinations too extensive and tiring for the participants and (b) all residents regardless of cognitive impairment level could have participated, which was not possible due to ethical considerations. Including all participants would have been of particular interest since cognitive impairment is highly associated with mortality^49^ and dysphagia and loss of dietary intake is more prominent in people with moderate to severe cognitive impairment.^50^, ^51^ The study design should also be considered. To investigate the causal effects of potential risk factors and predictors for malnutrition, longitudinal studies are required.

## 5 CONCLUSION

Mortality rate was higher among malnourished people than those who were well nourished. With a moderate to small effect size, severe dysphagia, poor oral health and female gender was predictors of malnutrition among older people in short‐term care. Together with earlier studies, these data demonstrate that management and identification of malnutrition, poor oral health and dysphagia should be given more attention in nursing care, including short‐term care. Future longitudinal studies are warranted to investigate the effects of potential risk factors on malnutrition.

## 6 CLINICAL RELEVANCE

### 6.1 Scientific rationale for study

Previous research has revealed that malnutrition among older people in nursing homes is common and can increase the risk of a number of negative clinical health outcomes. This study contributes with knowledge about associations between malnutrition, poor oral health, severe dysphagia and mortality among people in short‐term care.

### 6.2 Principal findings

Our results confirmed association between malnutrition, poor oral health, severe dysphagia and mortality.

### 6.3 Practical implications

Based on the result of this study it is important to identify older people at risk of malnutrition, dysphagia and poor oral health in short‐term care for adequate management and intervention.

## AUTHOR CONTRIBUTIONS

All authors contributed to the conception and design of the work. All authors contributed to the acquisition of data. PH was responsible for the statistical analyses. PH and SL drafted the first version of the manuscript. All authors critically revised the manuscript for important intellectual content. All authors gave final approval for publication.

## FUNDING INFORMATION

This study was supported by the Region Örebro County [RFR‐308581, RFR‐379081 and RFR‐475251]; Forte: The Swedish Research Council for Health, Working Life and Welfare [2013‐2127]; The Kamprad Family Foundation for Entrepreneurship, Research & Charity [20132115]; Swedish Society for Clinical Nutrition; and the Kempe Foundations. The sponsors did not play any role in the study. Open access funding provided by Umeå University.

## CONFLICT OF INTEREST STATEMENT

The authors declare that they have no competing interests.

## Data Availability

The dataset used and analysed during the current study are available from the corresponding author on reasonable request.
